# An Unprecedented Medium-Chain Diunsaturated *N*-acylhomoserine Lactone from Marine *Roseobacter* Group Bacteria

**DOI:** 10.3390/md17010020

**Published:** 2018-12-31

**Authors:** Lisa Ziesche, Laura Wolter, Hui Wang, Thorsten Brinkhoff, Marion Pohlner, Bert Engelen, Irene Wagner-Döbler, Stefan Schulz

**Affiliations:** 1 Institute of Organic Chemistry, Technische Universität Braunschweig, Hagenring 30, 38106 Braunschweig, Germany; l.ziesche@tu-braunschweig.de; 2 Institute for Chemistry and Biology of the Marine Environment, University of Oldenburg, Carl-von-Ossietzky-Straße 9-11, 26111 Oldenburg, Germany; laura.wolter@uni-oldenburg.de (L.W.); thorsten.brinkhoff@icbm.de (T.B.); marion.pohlner@uni-oldenburg.de (M.P.); bert.engelen@uni-oldenburg.de (B.E.); 3 Helmholtz Centre for Infection Research, Department of Medical Microbiology, Group Microbial Communication, Inhoffenstr. 7, 38124 Braunschweig, Germany; Hui.Wang@helmholtz-hzi.de (H.W.); Irene.Wagner-Doebler@helmholtz-hzi.de (I.W.-D.)

**Keywords:** quorum-sensing, structure elucidation, Rhodobacteraceae, autoinducers, bacterial signaling, Heck coupling, mass spectrometry, gas chromatography, sediment, AHL

## Abstract

*N*-acylhomoserine lactones (AHLs), bacterial signaling compounds involved in quorum-sensing, are a structurally diverse group of compounds. We describe here the identification, synthesis, occurrence and biological activity of a new AHL, *N*-((2*E*,5*Z*)-2,5-dodecadienoyl)homoserine lactone (**11**) and its isomer *N*-((3*E*,5*Z*)-3,5-dodecadienoyl)homoserine lactone (**13**), occurring in several *Roseobacter* group bacteria (Rhodobacteraceae). The analysis of 26 strains revealed the presence of **11** and **13** in six of them originating from the surface of the macroalgae *Fucus spiralis* or sediments from the North Sea. In addition, 18 other AHLs were detected in 12 strains. Compound identification was performed by GC/MS. Mass spectral analysis revealed a diunsaturated C_12_ homoserine lactone as structural element of the new AHL. Synthesis of three likely candidate compounds, **11**, **13** and *N*-((2*E*,4*E*)-2,4-dodecadienoyl)homoserine lactone (**5**), revealed the former to be the natural AHLs. Bioactivity test with quorum-sensing reporter strains showed high activity of all three compounds. Therefore, the configuration and stereochemistry of the double bonds in the acyl chain seemed to be unimportant for the activity, although the chains have largely different shapes, solely the chain length determining activity. In combination with previous results with other *Roseobacter* group bacteria, we could show that there is wide variance between AHL composition within the strains. Furthermore, no association of certain AHLs with different habitats like macroalgal surfaces or sediment could be detected.

## 1. Introduction

*N*-acylhomoserine lactones (AHLs) are well known signalling compounds used by Gram-negative bacteria for quorum-sensing (QS)-driven cell-to-cell communication. QS is a cell density-dependant mechanism to regulate physiological traits like antibiotic production, cell differentiation or biofilm formation [1,2,3,4,5,6,7]. AHLs constitute a γ-lactone ring and an acyl side chain that is usually even-numbered and unbranched, ranging in chain length from C_4_–C_18_ [3]. The stereochemistry of the lactone ring is *S*. Functional groups like hydroxy- or carbonyl-group can be present at C-3 of the acyl chain. The double bond of unsaturated AHLs is *Z*-configured and in the position ω-7, with few exceptions [3]. An additional double bond can occur at C-2 in *E*-configuration, a feature especially occurring in AHLs produced by marine *Roseobacter* group bacteria (Rhodobacteraceae). Roseobacters are abundant in the ocean, occurring in diverse habitats [8], e. g. in open waters, shore environments, sediments, attached to biotic and abiotic surfaces as well as in symbiosis with higher organisms like algae [9,10,11]. AHLs of this bacterial group have saturated, unsaturated and sometimes oxygenated acyl chains, ranging in length between C_8_ and C_18_ [12] with the exception of aromatic *p*-coumaroylhomoserine lactone produced by *Ruegeria pomeroyi* DSS-3 [13]. They are involved in various biological traits [14], e.g., in the production of the antibiotic tropodithietic acid in *Phaeobacter inhibens* [5] or cell differentiation in *Dinoroseobacter shibae* [4].

In a broader program, we currently look into the inventory of AHLs occurring in *Roseobacter* group bacteria. A non-targeted analytical approach was developed using extraction of AHLs from bacterial cultures by XAD-16 adsorption, solvent extraction, and direct analysis by GC/MS. This approach combines high-sensitivity with unbiased analysis and allows structural proposals to be made basing on the information rich EI-mass spectra obtained. We could successfully use this approach to identify several previously unknown AHLs [12,15,16] as well as related *N*-acylalanine methyl esters (NAMEs) [17,18]. We have previously analyzed AHLs from roseobacters isolated from macroalgae surfaces and found a high proportion of strains producing AHLs in various mixtures [12]. A specific AHL signature of macroalgae associated strains was not observed. In an extension of this study we investigated 16 strains obtained from one location of samples of the algae *Fucus spiralis*, collected from a single location (Neuharlingersiel, German Wadden Sea), nine strains from Norwegian trench sediments [19] and one sea water strain from the German/Danish coast to test for the occurrence of specific AHL signatures. During this investigation we detected two previously unreported AHLs, their identification, synthesis, and biological activities being reported.

## 2. Results

### 2.1. Occurrence of N-acylhomoserine Lactones in Roseobacter Group Bacteria of Fucus Spiralis and the Eastern North Sea

Sixteen roseobacters originating from *Fucus spiralis* from the German Wadden Sea and 10 strains from the eastern North Sea were cultivated in marine broth and analyzed for the presence of AHLs by GC/MS as described previously [12,20]. AHLs were detected in eight of the isolates from *F. spiralis* (50%) and three of the sediment strains (33%) as well as in the open water strain (Table 1). The highest numbers of individual compounds were detected on the extracts obtained from *Octadecabacter* sp. (Lw-22) and *Loktanella* sp. (D15 (40)), 12 and 13 AHLs being detected, exhibiting a distinct qualitative profile. The sediment strain *Phaeobacter* sp. (SK040) contained nine different AHLs, while the other sediment strains SK013 and SK032 had only one or two AHLs, comparable to the water column strain. The AHL composition of other strains varied between single compounds and mixtures (Table 1).

Octadecenoylhomoserine lactone (C18:1-HSL) and C16:1-HSL were the most common compounds, produced by six of the 12 bacteria. *Roseovarius sp*. D12-1.68 displayed a unique profile due to the presence of C12:0 as major AHL with a relatively short chain length compared to most other major AHLs of roseobacters [12,15,21]. *Dinoroseobacter shibae* MDLw-58 produced three different isomers of the C18:2-HSL, consistent with previous observations of closely related strains [20]. While the major component was 2*E*,11*Z*-C18:2-HSL, the location and configuration of the double bonds in the other two isomers remains unknown. Other diunsaturated AHLs with shorter chain lengths, rarely observed in strains taxonomically distant from roseobacters, occurred as well. These include C16:2-HSL in *Huaishuia* sp. SK032, C14:2-HSL in *Octadecabacter* sp. Lw-22 and several strains containing C12:2-HSL. Because this AHL was not previously reported and due to its abundant occurrence in the investigated strains, we determined its structure of this new AHL, as reported in Section 2.2. Less abundant were the oxygenated AHLs, 3-OH-C10- and 3-OH-C14-HSL. The only odd numbered AHLs were C15:0-HSL, C15:1-HSL and C17:1-HSL which occurred as minor components in several strains.

### 2.2. Identification and Synthesis of New Diunsaturated N-acylhomoserine Lactones from the Roseobacter Group

**Figure 1:**
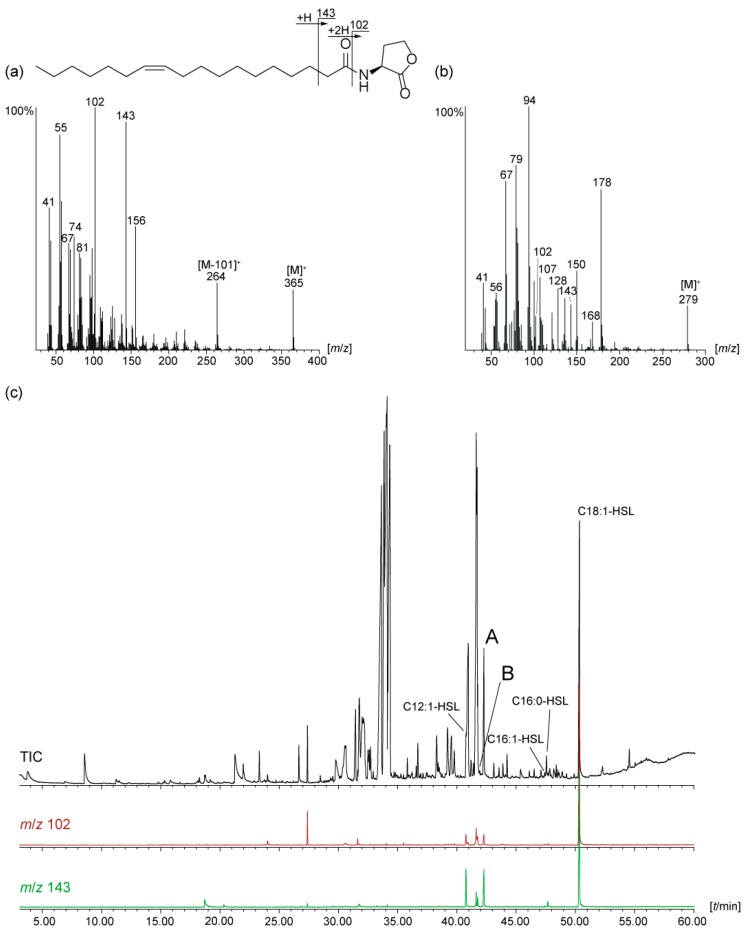
(**a**) Mass spectrum and fragmentation pattern of *N*-((Z)-11-octadecenoyl)homoserine lactone (*Z*11-C18:1-HSL); (**b**) mass spectrum of the unknown AHL **A**; (**c**) total ion chromatogram of the natural extract of *Octadecabacter* D4 (50) and characteristic ion traces *m*/*z* 102 and 143, indicating potential presence of AHLs.

During these analyses two compounds, **A** as the major component and **B** in lower concentration, were detected in five of the strains, whose mass spectra showed similarity to those of other AHLs. The spectra of A (Figure 1b) and **B** (Supplementary Figure S1) were very similar, although the quality of the spectra was often low due to overlapping peaks from other compounds. To elucidate their structure, analysis of mass spectral data and total synthesis were performed. 

Electron impact mass spectra of AHLs have a typical fragmentation pattern shown in Figure 1a. AHLs are characterized by the fragment ions *m*/*z* 102, 143 and a small [M]^+^ [15,22]. The ion *m*/*z* 102 is formed by α-cleavage of the homoserine lactone unit and transfer of two hydrogens, while McLafferty-rearrangement forms the ion *m*/*z* 143. The intensity of *m*/*z* 102 is higher in monounsaturated compared to diunsaturated AHLs and the intensities of *m*/*z* 102 and 143 decrease with the acyl chain length [15]. Cleavage of the homoserine moiety explains the ion [M--101]^+^, *m*/*z* 264, in the spectrum of (*Z*)-11-octadecenoylhomoserine lactone (Z11-C18:1-HSL, Figure 1a).

Compound **A** and **B** showed both ions *m*/*z* 102 and 143 and a putative [M]^+^ at *m/z* 279, indicating to be C12:2-homoserine lactones (C12:2-HSL). Additional ions at *m*/*z* 94 and 107, untypical for AHLs, were present in high intensity. The gas chromatographic retention index of **A** was 2422 and that of **B** 2388. High resolution ESI-MS of **A** delivered an ion at *m*/*z* 280.19071 [M + H]^+^, consistent with the formula C_16_H_26_NO_3_ (calc. 280.19072) required for an diunsaturated AHL. The homoserine lactone unit is indicated by the ion *m/z* 102.05496 (C_4_H_8_NO_2_) and the acyl chain by the ion *m/z* 179.14314 (C_12_H_19_O) in the ESI spectrum [18]. The location of the double bonds could not be determined with dimethyl disulfide derivatization [12,20] because of the low concentration of the compound. Nevertheless, the usual ω-7 position of double bonds in AHLs and the previous detection of *Z*5-C12:1-HSL in roseobacters [12] suggested **A** to be 2*E*,5*Z*-C12:2-HSL, because the second double-bond in all known natural AHLs is located at C-2 with *E*-configuration. The close proximity of the double bonds might also favor a double bond shift into conjugation during biosynthesis with concomitant double bond isomerization, leading to 2*E*,4*E*-C12:2-HSL. Furthermore, deconjugation is a known process known to occur during formation of α,β-unsaturated amides under basic conditions, leading potentially to 3*E*,5*Z*-C12:2-HSL [23,24]. Therefore, we decided to synthesize all three compounds to reveal insight into the MS and GC behavior of the isomers and also allowing to perform bioassays to investigate whether slight changes in double bond location and geometry have an influence on the activity in AHL reporter assays.

**Scheme 1:**
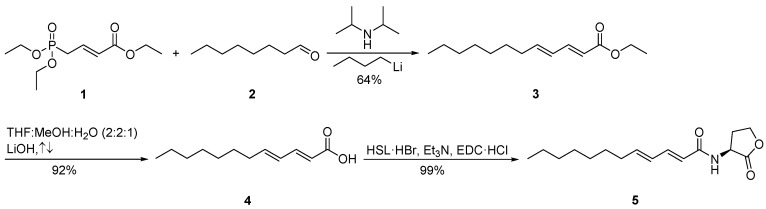
Synthesis of *N*-((2*E*,4*E*)-2,4-dodecadienoyl)homoserine lactone (2*E*,4*E*-C12:2-HSL, **5**). l-homoserine lactone hydrobromide (HSL·HBr), *N*-(3-dimethylaminopropyl)-*N*’-ethylcarbodiimide hydrochloride (EDC·HCl).

The synthesis of 2*E*,4*E*-C12:2-HSL (**5**) started with a homologous Horner-Wadsworth-Emmons reaction of triethyl (*E*)-4-phosphonocrotonate (**1**) with octanal (**2**) to furnish ethyl (2*E*,4*E*)-2,4-dodecadienoate (**3**) (Scheme 1). After saponification with lithium hydroxide, **4** was coupled with l-homoserine lactone hydrobromide (HSL·HBr) in the presence of *N*-(3-dimethylaminopropyl)-*N*′-ethylcarbodiimide hydrochloride (EDC·HCl) and triethylamine, yielding the desired AHL *N*-((2*E*,4*E*)-2,4-dodecadienoyl)homoserine lactone (**5**).

**Scheme 2:**
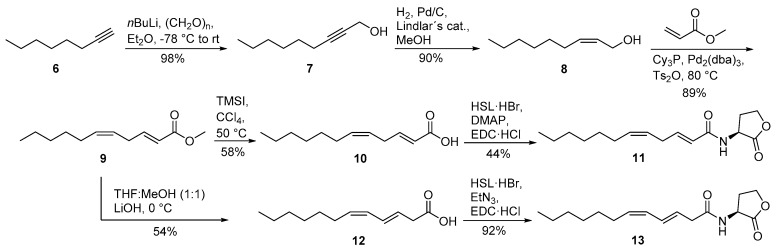
Synthesis of *N*-((2*E*,5*Z*)-2,5-dodecadienoyl)homoserine lactone (2*E*,5*Z*-C12:2-HSL, **11**) and *N*-((3*E*,5*Z*)-3,5-dodecadienoyl)homoserine lactone (3*E*,5*Z*-C12:2-HSL, **13**). Tricyclohexylphosphine (Cy_3_P), tris(dibenzylideneacetone)dipalladium (Pd_2_(dba)_3_), l-homoserine lactone hydrobromide (HSL·HBr), *N*-(3-dimethylaminopropyl)-*N*’-ethylcarbodiimide hydrochloride (EDC·HCl), *p*-dimethylaminopyridine (DMAP).

The isomers 2*E*,5*Z*-C12:2-HSL (**11**) and 3*E*,5*Z*-C12:2-HSL (**13**) were synthesized from octyne (**6**) (Scheme 2). The addition of paraformaldehyde furnished non-2-yn-1-ol (**7**). The allylic alcohol (*Z*)-non-2-en-1-ol (**8**) was obtained after hydrogenation with Lindlar′s catalysts. A Heck reaction according to Tsukuda et al. [25] using methyl acrylate instead of butyl acrylate yielded in excellent yield the desired diastereomerically pure methyl (2*E*,5*Z*)-2,5-dodecadienoate (**9**) that was saponified with lithium hydroxide. The basic conditions lead to a rearrangement of the double bond from C-2 to C-3, forming acid **12**. This acid was coupled with HSL·HBr in the presence of EDC·HCl and triethylamine to form *N*-((3*E*,5*Z*)-3,5-dodecadienoyl)homoserine lactone (**13**). The saponification of ester **9** can also be performed under milder conditions, thus preventing the rearrangement. First, ester **9** was transesterified to the trimethylsilyl ester with trimethylsilyl iodide (TMSI) in CCl_4_. Aqueous hydrolysis delivers acid **10** without rearrangement [26]. Finally, acid **10** was coupled with HSL·HBr in the presence of EDC·HCl and *p*-dimethylaminopyridine (DMAP) to furnish *N*-((2*E*,5*Z*)-2,5-dodecadienoyl)homoserine lactone (**11**). The isomerization of the double bond, leading again to **13**, can also be induced by the use of triethylamine in this step instead of the weaker base DMAP [24]. AHL **11** is not stable for prolonged storage under room temperature, leading to the isomerized compound **13** and other stereoisomers.

**Figure 2:**
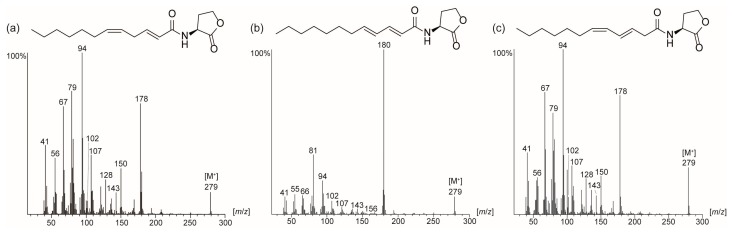
Mass spectra of synthetic AHLs: (**a**) 2*E*,5*Z*-C12:2-HSL (**11**); (**b**) 2*E*,4*E*-C12:2-HSL (**5**); (**c**) 3*E*,5*Z*-C12:2-HSL (**13**).

Comparison of both mass spectra (Figure 2) and gas chromatographic retention indices of the synthesized AHLs with those of the natural compounds indicated the unknown major AHL **A** to be 2*E*,5*Z*-C12:2-HSL (**11**), while the minor component **B** is its rearrangement product, 3*E*,5*Z*-C12:2-HSL (**13**). The mass spectrum of **5** differs from those of **11** and **13**, showing an ion at *m*/*z* 180 instead of *m*/*z* 178. The ions *m*/*z* 94, 102, 107 and 143 are present, but the intensity of these fragment ions is different to those of the natural compounds, as is the retention index of 2510. HR-MS data showed that the ions *m*/*z* 107 and 94, not observed in high intensity in other diunsaturated AHLs with longer acyl chains, are composed of C_8_H_11_ and C_6_H_6_O, respectively. They seem to be formed by acyl cleavage followed by allylic chain cleavage with or without previous elimination of water. 

### 2.3. Activity of N-acylhomoserine Lactones in AHL Reporter Assays

The three C12:2-HSLs **5**, **11**, and **13** as well as 2*E*,11*Z*-C18:2-HSL, a major AHL of the *Roseobacter* group model strain *Dinoroseobacter shibae* DFL12 [4,20] were tested for quorum sensing activity with two sensor strains (Table 2). *Escherichia coli* MT102 (pJBA132) [27] responds primarily to short chain AHLs while *Pseudomonas putida* F117 (pRK-C12) [28] prefers long chain AHLs. Both strains do not produce AHLs but are able to respond to them through the expression of a LuxR-controlled promoter fused to a gene coding for an easily detectable output signal, fluorescence [15]. In addition, C6:0-, C8:0- and 3-oxo-C8:0-HSL for *E.coli* MT102 and Z9-C16:1-, 3-oxo-C8:0- and 3-oxo-C12:0-HSL for *P. putida* F117 were tested as references. The maximum fold induction with sensor strain *E.coli* MT102 was low for all target AHLs, **5**, **11**, **13** as well as 2*E*,11*Z*-C18:2-HSL, but C6 and C8-HSLs showed activity as expected.

In contrast, sensor *P. putida* F117, most sensitive to C12:0-HSL [16], showed high induction upon exposure to AHLs **5**, **11**, and **13,** as well as 3-oxo-C12:0-HSL and Z9-C16:1-HSL, a lower activity for 3-oxo-C8:0-HSL being noted. The almost identical values exhibited by the four C12 compounds evidence a certain degree of selectivity dependent on chain length, but the reporter strain is insensitive to position and configuration of the double bonds, as well as presence or absence of a 3-oxo functional group.

## 3. Discussion

The unknown AHL **A** was identified to be 2*E*,5*Z*-C12:2-HSL (**11**). This new AHL is the smallest AHL with two double-bonds identified so far and occurs in several roseobacters. The minor 3*E*,5*Z*-C12:2-HSL (**13**) seems to be a rearrangement product of **11**. Although we cannot exclude that the process took place during work-up, the observed chemical instability of **11** may point to the formation of **13** as a bacterial metabolic product. Different roseobacters are able to produce a bishomologous series of diunsaturated AHLs with double-bonds at C-2 and the ω-7 position [12,15,16,20]. AHL **11** extends this series to a shorter C_12_ chain length. It is also within the preferred chain-length of major AHLs of roseobacters that ranges from C_12_ to C_18_, with the exception of 3-OH-C10-AHL. Although we analyzed many roseobacters for AHL presence using GC/MS as well as HPLC/MS methods, we never found evidence for the presence of *p*-coumaroyl-HSL reported from *Ruegeria pomeroyi* DSS-3 [13] in any of these strains.

Although the AHLs **5**, **11** and **13** have the same chain length, the shape of the side chain differs. The two (*E*)-configured double bonds of 2*E*,4*E*-C12:2-HSL lead to a straight aliphatic chain, while the (*Z*)-configured double-bonds in 2*E*,5*Z*-C12:2-HSL and 3*E*,5*Z*-C12:2-HSL induce a bend in the chain. 3-oxo-C12:0-HSL even has a bend chain due to H-bonding towards the amide carbonyl group [29]. Obviously, there is no influence of the chain configuration on the activity of the reporter strain. Therefore, the configuration of the side chain does not seem to be recognized by the receptor, although the chain-length obviously is. Whether this is also true for the cognate receptors in the roseobacters is unknown. Nevertheless, the (2*E*)-double bond is not common in fatty acids of roseobacters [30] and thus the prominent occurrence of this structural motif and of diunsaturated AHLs in general indicate the importance of the double bonds for their function as signaling compounds of these bacteria.

No general association of specific AHLs or their mixtures with certain habitats was observed. This includes strains originating from the same host organism as *F. spiralis* reported here, but also strains from the sediment or the water phase [12,15]. In general, surface-associated strains seem to produce AHLs more often under laboratory conditions compared to those obtained from sediments or the water column.

## 4. Materials and Methods

### 4.1. General Experimental Procedures

General conditions: Chemicals were obtained from Sigma-Aldrich (Taufkirchen, Germany), Carl Roth (Karlsruhe, Germany) or from Acros Organics (Schwerte, Germany) and were used without further purification. Solvents were purified by distillation and dried according to standard procedures. Moisture- and/or oxygen-sensitive reactions were carried out under a nitrogen atmosphere in vacuum-heated flasks with dried solvents. Thin-layer chromatography (SiO_2_, TLC) was performed on 0.20 mm Macherey-Nagel silica gel plates (Polygram SIL G/UV254), and column chromatography was performed with Merck silica gel 60 (0.040–0.063 mm) by using standard flash chromatographic methods. NMR spectra were recorded on DRX-400 (400 MHz), AV III-400 (400 MHz) or AV II-600 (600 MHz) spectrometers (Bruker: Bremen, Germany), and were referenced against TMS (δ = 0.00 ppm), CDCl_3_ (δ = 7.26 ppm) for ^1^H-NMR and CDCl_3_ (δ = 77.01 ppm) for ^13^C-NMR experiments. GC/MS analyses of extracts were carried out on an GC 7890A gas chromatograph connected to a 5975C mass-selective detector (Agilent; Waldbronn, Germany). Synthetic samples were analyzed on an HP GC 6890 system connected to an HP 5973 mass selective detector fitted with a HP-5 MS fused-silica capillary column (30 m × 0.25 mm i.d., 0.22 µm film; (Agilent; Waldbronn, Germany). Conditions were as follows: carrier gas (He): 1.2 mL min^−1^; injection volume: 1 mL; injector: 250 °C; transfer line: 300 °C, EI 70 eV. The gas chromatograph was programmed as follows: 50 °C (5 min isothermal), increasing with 5 °C min^−1^ to 320 °C, and operated in splitless mode for XAD extracts and 50 °C (5 min isothermal), increasing with 10 °C min^−1^ to 320 °C in split mode (20:1) for synthetic compounds. Gas chromatographic retention indices, *I*, were determined from a homologous series of *n*-alkanes. Acids were transformed into volatile trimethylsilyl esters with *N*-methyl-*N*-(trimethylsilyl)trifluoroacetamide (MSTFA) for analysis by GC/MS [31]. HRMS analyses were carried out on a Thermo Fisher linear iontrap (Thermo Fisher: Bremen, Germany) coupled with an LTQ-Orbitrap mass spectrometer (Thermo Fisaher: Bremen, Germany) using ESI positive mode. ESI measurements were performed by direct infusion mode using a custom-made micro-spray device mounted on a Proxeon nano-spray ion source. All solvents used were of LC/MS grade.

### 4.2. Strains and Culture Conditions

The bacterial strains were collected at various occasions in the North Sea [19]. Information on bacterial strains investigated is shown in Table 3.

### 4.3. Bacteria Cultivation and XAD Extraction

Bacterial strains were inoculated from marine broth (MB) medium agar plates into a preculture (50 mL, MB medium) and were cultivated for one to three days at 20/28 °C and 160 rpm. Bacterial cultures (100 mL) were grown from precultures for three to five days in MB medium at 20/28 °C and 160 rpm containing Amberlite XAD-16 (2 g). The XAD-16 resin was cleaned using Soxhlet extraction with acetonitrile, methanol and finally diethyl ether. The adsorbent was separated from the culture by filtration and extracted three times with CH_2_Cl_2_/H_2_O (10:1). The combined organic phases were dried with MgSO_4_, and the solvent was removed under reduced pressure. The extract was dissolved in CH_2_Cl_2_ (50 µL) and analyzed by GC/MS [20].

### 4.4. AHL Reporter Assays

The sensor strain *Pseudomonas putida* pRK-C12 [27] was inoculated from plates into a preculture which was grown on LB medium (20 mL with 20 mg/mL gentamycin) at 30 °C with shaking (160 rpm) overnight. The next day fresh medium was added, and the culture was grown on a shaking platform for 1–2 h until an OD620 value of 1.0 was reached. For the test, LB medium (99 µL) and 1 µL of the test compound (final concentration 10 µM, stock solution 1 mg/mL in dichlormethane) were pipetted into 96-well microtiter plates, and the sensor strain (100 µL) was added. Microtiter plates were incubated at 30 °C and shaken. Fluorescence was determined in a Victor 1420 Multilabel Counter (Perkin Elmer; Rodgau, Germany) at an excitation wavelength of 485 nm and a detection wavelength of 535 nm every 60 min for the first 6 h, and finally after 24 h of incubation. The OD620 value was also measured. Dichlormethane was used as negative control, and synthetic 3-oxo-C12:0-HSL was used as positive control. Fold induction of fluorescence was calculated by dividing the specific fluorescence (gfp535/OD620) of the test sample by the specific fluorescence of the negative control. Mean and standard deviation of three biological replicas after 6 h were determined, because fluorescence decreased slightly in the 24 h time point. The sensor strain *Escherichia coli* MT102 [29] was used as described [15] and the highest values obtained after 24 h in this case are reported in Table 2.

### 4.5. Synthetic Procedures

#### 4.5.1. l-Homoserine Lactone Hydrobromide

A solution of bromoacetic acid (5.12 g, 36.9 mmol) and l-methionine (5.00 g, 33.5 mmol) in H_2_O/2-propanol/AcOH (5:5:2, 48.3 mL) was heated to reflux for 8 h. The solvent of the cooled mixture was evaporated. The orange solid was dissolved in dioxane/HCl (2:1, 20.1 mL), heated 10 min at 50 °C and stirred for 5 h at room temperature. The reaction mixture was placed in a fridge over night to evoke precipitation. l-Homoserine lactone hydrobromide was obtained by filtration and washed with cold isopropanol (3.19 g, 17.5 mmol, 52%) [35].

^1^H-NMR (400 MHz, DMSO-d_6_): δ = 4.44 (t, *J* = 8.8 Hz, 1H), 4.36–4.25 (m, 2H), 2.60–2.51 (m, 1H), 2.42–2.31 (m, 1H); ^13^C-NMR (100 MHz, DMSO-d_6_): δ = 173.4, 66.3, 47.8, 27.0; MS (70 eV, EI): *m*/*z* (%): 43 (100), 57 (90.5), 56 (62.4), 42 (40.1), 44 (16.0), 41 (8.3), 101 (5.0) [M^+^], 54 (4.8), 39 (4.3), 73 (4.0). 

#### 4.5.2. Ethyl (2*E*,4*E*)-2,4-Dodecadienoate (**3**)

*N*,*N*-Diisopropylamine (1.64 mL, 11.7 mmol) was dissolved in THF (25 mL) and cooled to −78 °C. *n*-Butyl lithium in hexane (1.6 M, 4.88 mL, 7.8 mmol) was added slowly to the mixture. The solution was stirred for 10 min at −78 °C, followed by 15 min at 0 °C. (*E*)-Triethyl-4-phosphonocrotonate (**1**, 1.30 mL, 5.8 mmol) was added slowly at −78 °C under stirring and after 15 min octanal (**2**, 0.61 mL, 3.9 mmol) was added at the same temperature, again stirring continued for 15 min. The reaction mixture was allowed to warm up to room temperature and stirred for 2.5 h. Sat. NH_4_Cl solution was added and the mixture was extracted three times with ethyl acetate. The combined organic phases were dried with MgSO_4_, filtered and the solvent was evaporated in *vacuo*. The crude product was purified by flash chromatography on silica [pentane/EtOAc (30:1)] to receive the desired product as a clear oil (280 mg, 1.25 mmol, 64%) [36].

R_f_ = 0.4 (pentane/EtOAc 30:1); ^1^H-NMR (400 MHz, CDCl_3_): δ = 7.29–7.22 (m, 1H), 6.20–6.08 (m, 2H), 5.78 (d, *J* = 15.4 Hz, 1H), 4.19 (q, *J* = 7.2 Hz, 2H), 2.16 (q, *J* = 7.0 Hz, 2H), 1.43 (quin, *J* = 7.3 Hz, 2H), 1.32–1.24 (m, 11H), 0.88 (t, *J* = 7.1 Hz, 3H); ^13^C-NMR (100 MHz, CDCl_3_): δ = 167.3, 145.1, 144.8, 128.3, 119.1, 60.1, 33.0, 31.8, 29.1, 29.1, 28.7, 22.6, 14.3, 14.1; MS (70 eV, EI): *m*/*z* (%): 125 (100), 97 (58.4), 81 (51.7), 67 (39.6), 179 (34.9), 98 (30.6), 79 (30.5), 95 (25.3), 127 (22.8), 99 (22.4), 224 (21.0) [M^+^].

#### 4.5.3. (2*E*,4*E*)-2,4-Dodecadienoic Acid (**4**)

Lithium hydroxide (106.8 mg, 4.46 mmol) was added to a stirred solution of ester **3** (50 mg, 0.22 mmol) in THF/MeOH/H_2_O (2:2:1) and stirred for 12 h under reflux. The mixture was acidified with 2 m sulfuric acid and extracted three times with dichloromethane. The combined organic phases were dried with Na_2_SO_4_, filtered and concentrated in *vacuo* to get the desired product after purification by flash chromatography on silica [pentane/EtOAc (2:1)] as a white solid (39.9 mg, 0.20 mmol, 92%).

R_f_ = 0.3 (pentane/EtOAc 2:1); ^1^H-NMR (400 MHz, CDCl_3_): δ = 7.35 (dd, *J* = 15.3, 10.0 Hz, 1H), 6.24–6.16 (m, 2H), 5.78 (d, *J* = 15.3 Hz, 1H), 2.18 (q, *J* = 7.3 Hz, 2H), 1.43 (quin, *J* = 7.3 Hz, 2H), 1.33–1.20 (m, 8H), 0.88 (t, *J* = 7.0 Hz, 3H); ^13^C-NMR (100 MHz, CDCl_3_): δ = 172.8, 147.5, 146.3, 128.2, 118.2, 33.1, 31.8, 29.1, 29.1, 28.6, 22.6, 14.1; MS (70 eV, EI, trimethylsilyl ester): *m*/*z* (%): 169 (100), 253 (64.0), 73 (31.9), 155 (30.1), 170 (20.5), 75 (17.5), 254 (15.7), 268 (13.8) [M^+^], 81 (13.4), 171 (9.2).

#### 4.5.4. *N*-((2*E*,4*E*)-2,4-Dodecadienoyl)homoserine Lactone (**5**)

l-Homoserine lactone hydrobromide (53 mg, 0.29 mmol) was dissolved in dry dichloromethane. Triethylamine (0.04 mL, 0.29 mmol) was added to the solution, followed by the addition of acid **4** (57 mg, 2.29 mmol) and EDC·HCl (56 mg, 0.29 mmol). The reaction mixture was stirred for 12 h at room temperature and washed with H_2_O, sat. NaHCO_3_ solution, and brine. The organic layers were extracted three times with dichloromethane, dried with Na_2_SO_4_, filtered and concentrated. The crude product was purified by flash chromatography on silica [pentane/EtOAc (1:1)] to obtain pure AHL **5** (80.6 mg, 0.28 mmol, 99%).

R_f_ = 0.3 (pentane/EtOAc 1:1); ^1^H-NMR (600 MHz, CDCl_3_): δ = 7.23 (dd, *J* = 15.0, 9.7 Hz, 1H), 6.25 (br s, 1H), 6.17–6.08 (m, 2H), 5.81 (d, *J* = 15.1 Hz, 1H), 4.66 (ddd, *J* = 11.6, 8.6, 6.0 Hz, 1H), 4.48 (td, *J* = 9.0, 1.1 Hz, 1H), 4.31 (ddd, *J* = 11.3, 9.3, 5.9 Hz, 1H), 2.87 (dddd, *J* = 12.5, 8.5, 5.9, 1.2 Hz, 1H), 2.22–2.18 (m, 1H), 2.15 (q, *J* = 7.0 Hz, 2H), 1.41 (quin, *J* = 7.0 Hz, 2H), 1.33–1.26 (m, 8H), 0.88 (t, *J* = 7.2 Hz, 3H); ^13^C-NMR (150 MHz, CDCl_3_): δ = 175.5, 166.6, 144.4, 142.7, 127.8, 119.9, 66.0, 49.2, 32.8, 31.5, 30.4, 28.9, 28.9, 28.5, 22.4, 14.0 (Supplementary Figure S4); HRMS (ESI+) *m/z*: 302.1728 [M + Na]^+^, calculated for C_16_H_25_NO_3_Na 302.1727 [M + Na]^+^.

#### 4.5.5. Non-2-yn-1-ol (**7**)

*n*-Butyl lithium in hexane (1.6 m, 31.25 mL, 50 mmol) was added at −78 °C to a stirred solution of 1-octyne (**6**, 5 g, 45 mmol) in dry diethyl ether and stirred for 30 min. Paraformaldehyde (2.77 g, 90 mmol) was added and the mixture was allowed to warm up to room temperature. Sat. NH_4_Cl solution was added, the phases were separated and extracted three times with diethyl ether. The combined organic phases were washed with brine, dried with Na_2_SO_4_, filtered and the solvent was evaporated. The crude product was purified by flash chromatography on silica [pentane/EtOAc (7:1)] to furnish the desired product as a clear oil (6.2 g, 40 mmol, 98%) [37].

R_f_ = 0.4 (pentane/EtOAc 7:1); ^1^H-NMR (400 MHz, CDCl_3_): δ = 4.25 (dt, *J* = 5.8, 2.1 Hz, 2H), 2.21 (tt, *J* = 7.1, 2.2 Hz, 2H), 1.51 (quin, *J* = 7.3 Hz, 2H), 1.41–1.24 (m, 6H), 0.89 (t, *J* = 6.8 Hz, 3H); ^13^C-NMR (100 MHz, CDCl_3_): δ = 86.7, 78.3, 51.4, 31.3, 28.6, 28.5, 22.5, 18.7, 14.0; MS (70 eV, EI): *m*/*z* (%): 67 (100), 55 (97.2), 41 (95.0), 93 (73.4), 70 (72.5), 39 (68.8), 43 (64.0), 69 (58.0), 79 (57.4), 83 (56.5), 122 (10.0) [M^+^-H_2_O].

#### 4.5.6. Non-2-en-1-ol (**8**)

Lindlar’s catalyst (50 mg) was added to a solution of ynol **7** (500 mg, 3.57 mmol) and methanol (10 mL). The mixture was stirred for 20 min at room temperature under a H_2_ atmosphere. The catalyst was filtered through a short pad of silica and the solvent was evaporated. The product was received after purification by flash chromatography on silica [pentane/EtOAc (10:1)] as a colorless oil (454 mg, 3.19 mmol, 90%). 

R_f_ = 0.3 (pentane/EtOAc 10:1); ^1^H-NMR (400 MHz, CDCl_3_): δ = 5.63–5.51 (m, 2H), 4.2 (d, *J* = 6.0 Hz, 2H), 2.07 (q, *J* = 6.7 Hz, 2H), 1.40–1.23 (m, 8H), 0.88 (t, *J* = 7.0 Hz, 3H); ^13^C-NMR (100 MHz, CDCl_3_): δ = 133.3, 128.3, 58.6, 31.7, 29.6, 28.9, 27.4, 22.6, 14.1; MS (70 eV, EI): *m*/*z* (%): 57 (100), 41 (69.6), 55 (50.7), 43 (49.0), 54 (41.0), 67 (39.4), 82 (38.7), 81 (36.0), 68 (34.6), 95 (33.7), 124 (22.7) [M^+^-H_2_O], 142 (0.3) [M^+^].

#### 4.5.7. Methyl (2*E*,5*Z*)-2,5-Dodecadienoate (**9**)

The allylic alcohol **8** (454 mg, 3.19 mmol) was added to a mixture of tris(dibenzylideneacetone)dipalladium (83 mg, 0.08 mmol), tricyclohexylphosphine (45 mg, 0.16 mmol) and *p*-toluenesulfonic anhydride (1.25 g, 3.8 mmol) in methyl acrylate (16 mL) and stirred for 12 h at 80 °C in a special high-pressure vial under argon. The catalyst was filtered through a short silica column and washed with diethyl ether. The solvent was evaporated in *vacuo* and the crude product was purified by flash chromatography on silica [pentane/EtOAc (30:1)] to the desired product (599.7 mg, 2.85 mmol, 89%) [25].

R_f_ = 0.4 (pentane/EtOAc 30:1); ^1^H-NMR (400 MHz, CDCl_3_): δ = 6.97 (dt, *J* = 15.7, 6.4 Hz, 1H), 5.82 (dt, *J* = 15.7, 1.8 Hz, 1H), 5.56-5.34 (m, 2H), 3.73 (s, 3H), 2.88 (dd, *J* = 6.9, 6.3 Hz, 2H), 2.01 (q, *J* = 6.6 Hz, 2H), 1.38–1.26 (m, 8H), 0.88 (t, *J* = 7.1 Hz, 3H); ^13^C-NMR (100 MHz, CDCl_3_): δ = 167.1, 148.1, 133.8, 124.9, 121.1, 51.4, 35.1, 32.6, 31.7, 29.3, 28.8, 22.6, 14.1; MS (70 eV, EI): *m*/*z* (%): 79 (100), 111 (90.3), 81 (70.2), 67 (63.6), 41 (60.0), 210 (52.8) [M^+^], 55 (50.0), 95 (47.4), 80 (47.3), 100 (45.9).

#### 4.5.8. (2*E*,5*Z*)-2,5-Dodecadienoic Acid (**10**)

Trimethylsilyl iodide (0.038 mL, 0.268 mmol) was added to a stirred solution of ester **9** (30 mg, 0.134 mmol) in 0.5 mL CCl_4_ and warmed to 50 °C for one hour. The mixture was washed with sat. NH_4_Cl solution followed by sat. sodium thiosulfate solution and extracted three times with dichloromethane. The combined organic phases were dried with MgSO_4_, filtered and concentrated in *vacuo* to get the desired product after purification by flash chromatography on silica [pentane/Et_2_O (5:1)] as a white solid (15 mg, 0.077 mmol, 58%).

R_f_ = 0.2 (pentane/EtOAc 5:1); ^1^H-NMR (400 MHz, CDCl_3_): δ = 7.08–7.01(m, 1H), 5.81 (d, *J* = 15.6 Hz, 1H), 5.42–5.26 (m, 2H), 2.94 (dd, *J* = 6.9, 6.3 Hz, 2H), 2.01 (q, *J* = 6.9 Hz, 2H), 1.35–1.24 (m, 8H), 0.81 (t, *J* = 7.0 Hz, 3H); ^13^C-NMR (100 MHz, CDCl_3_): δ = 172.8, 148.8, 133.4, 124.4, 121.5, 33.0, 28.9, 28.8, 28.7, 27.8, 22.6, 14.0; MS (70 eV, EI, trimethylsilyl ester): *m*/*z* (%): 169 (100), 253 (42.4), 73 (40.2), 155 (21.9), 75 (19.9), 170 (15.1), 81 (13.5), 254 (8.8), 43 (8.7), 268 (8.0) [M^+^].

#### 4.5.9. *N*-((2*E*,5*Z*)-2,5-Dodecadienoyl)homoserine Lactone (**11**)

l-Homoserine lactone hydrobromide (22 mg, 0.1224 mmol) was dissolved in dry dichloromethane, followed by the addition of *p*-dimethylaminopyridine (DMAP) (15 mg, 0.1224 mmol) and acid **10** (24 mg, 0.1224 mmol). EDC·HCl (23.5 mg, 0.1224 mmol) was added at 0 °C, the solution was stirred for 5 min at 0 °C and for 12 h at room temperature. The reaction mixture was washed one time with H_2_O, sat. NaHCO_3_ solution and brine. The organic layers were extracted three times with dichloromethane, dried with Na_2_SO_4_, filtered and the solvent was evaporated. The crude product was purified by flash chromatography on silica [pentane/EtOAc (1:1)] to the desired AHL (15 mg, 0.054 mmol, 44%).

R_f_ = 0.2 (pentane/EtOAc 1:1); ^1^H-NMR (600 MHz, CDCl_3_): δ = 6.91 (dt, *J* = 15.4, 6.4 Hz, 1H), 6.06 (br s, 1H), 5.83 (dt, *J* = 15.3, 1.7 Hz, 1H), 5.53–5.36 (m, 2H), 4.62 (ddd, *J* = 11.6, 8.6, 5.6 Hz, 1H), 4.50–4.47 (m, 1H), 4.33–4.28 (m, 1H), 2.92–2.87 (m, 3H), 2.20–2.12 (m, 1H), 2.01 (q, *J* = 7.0 Hz, 2H), 1.39–1.25 (m, 8H), 0.88 (t, *J* = 7.0 Hz, 3H); ^13^C-NMR (150 MHz, CDCl_3_): δ = 175.5, 166.3, 145.1, 133.8, 125.0, 122.5, 66.2, 49.4, 34.9, 32.6, 31.7, 30.7, 29.3, 28.9, 22.6, 14.1 (Supplementary Figure S2); HRMS (ESI+) *m/z*: 280.1909 [M + H]^+^, calculated for C_16_H_26_NO_3_ 280.1907 [M + H]^+^; 302.1729 [M + Na]^+^, calculated for C_16_H_25_NO_3_Na 302.1727 [M + Na]^+^.

#### 4.5.10. (3*E*,5*Z*)-3,5-Dodecadienoic Acid (**12**)

Lithium hydroxide (0.238 mL, 1.5 M) was added to a stirred solution of ester **9** (50 mg, 0.238 mmol) in THF/MeOH (1:1) and stirred for 3 h at 0 °C. The mixture was acidified with 1 m HCl and extracted three times with dichloromethane. The combined organic phases were dried with Na_2_SO_4_, filtered and concentrated in *vacuo* to get the desired product after purification by flash chromatography on silica [pentane/EtOAc (5:1)] as a white solid (25 mg, 0.128 mmol, 54%).

R_f_ = 0.2 (pentane/EtOAc 5:1); ^1^H-NMR (400 MHz, CDCl_3_): δ = 6.19–5.98 (m, 2H), 5.72 (dt, *J* = 15.1, 7.1 Hz, 1H), 5.39 (dt, *J* = 10.8, 7.6 Hz, 1H), 3.10 (d, *J* = 7.2 Hz, 2H), 2.09 (q, *J* = 7.6, 7.2 Hz, 2H), 1.43–1.26 (m, 8H), 0.86 (t, *J* = 6.9 Hz, 3H); ^13^C-NMR (100 MHz, CDCl_3_): δ = 178.2, 135.4, 134.7, 129.3, 121.3, 37.7, 31.7, 29.2, 28.9, 27.8, 22.6, 14.1; MS (70 eV, EI, trimethylsilyl ester): *m*/*z* (%): 73 (100), 75 (23.5), 253 (15.2), 74 (14.4), 268 (12.2) [M^+^], 79 (10.9), 150 (10.9), 41 (6.4), 67 (6.4), 224 (6.4).

#### 4.5.11. *N*-((3*E*,5*Z*)-3,5-Dodecadienoyl)homoserine Lactone (**13**)

l-Homoserine lactone hydrobromide (21.8 mg, 0.12 mmol) was dissolved in dry dichloromethane. Triethylamine (0.02 mL, 0.12 mmol) was added to the solution, followed by the addition of acid **12** (24.4 mg, 0.12 mmol) and EDC·HCl (23 mg, 0.12 mmol). The reaction mixture was stirred for 12 h at room temperature and washed successively with H_2_O, sat. NaHCO_3_ solution and brine. The organic layers were extracted three times with dichloromethane, dried with Na_2_SO_4_, filtered and the solvent was evaporated. The crude product was purified by flash chromatography on silica [pentane/EtOAc (1:1)] to obtain the desired AHL **13** (32 mg, 0.115 mmol, 92%). 

R_f_ = 0.2 (pentane/EtOAc 1:1); ^1^H-NMR (400 MHz, CDCl_3_): δ = 6.34 (br s, 1H), 6.12–5.93 (m, 2H), 5.66–5.51 (m, 2H), 4.51 (ddd, *J* = 11.5, 8.5, 6.5 Hz, 1H), 4.41–4.36 (m, 1H), 4.2 (ddd, *J* = 11.0, 9.3, 6.0 Hz, 1H), 3.00 (d, *J* = 7.3 Hz, 2H), 2.74–2.68 (m, 1H), 2.15–2.04 (m, 1H), 2.00 (q, *J* = 7.2 Hz, 2H), 1.32–1.20 (m, 8H), 0.81 (t, *J* = 7.0 Hz, 3H); ^13^C-NMR (100 MHz, CDCl_3_): δ = 175.4, 171.6, 135.8, 135.7, 129.2, 122.0, 66.0, 49.2, 39.9, 32.6, 31.7, 30.1, 29.1, 28.8, 22.5, 14.0 (Supplementary Figure S3); HRMS (ESIs1*/z*: 280.1909 [M + H]^+^, calculated for C_16_H_26_NO_3_ 280.1907 [M + H]^+^; 302.1729 [M + Na]^+^, calculated for C_16_H_25_NO_3_Na 302.1727 [M + Na]^+^.

## Figures and Tables

**Table 1:** AHL production of *Roseobacter* group isolates from *Fucus spiralis* (German Wadden Sea) and sediments as well as open waters (Eastern North Sea) ^a^.

Strain	Genus Affiliation	C12:0	C12:1	C12:2 (11)	C12:2 (13)	3-OH-C10	3-OH-C14	C14:0	C14:1	C14:2	C15:0	C15:1	C16:0	C16:1	C16:2	C17:1	C18:0	C18:1	C18:2	C18:2	C18:2
*Fucus Spiralis*																					
D12-1.68	*Roseovarius* sp.	94.4							1.3			4.3									
D3	*Loktanella* sp.			15.7	2.4	21.2	60.7														
Lw-22	*Octadecabacter* sp.		6.6	3.7	0.7			4.6	72.7	4.3	1.2	1.9	0.9	0.6			1.1	1.8			
D4 (50)	*Octadecabacter* sp.		8.3	25.8	4.4								1.1	0.7				59.8			
Lw-26b	*Loktanella* sp.						100														
D15 (40)	*Loktanella* sp.		3.9	5.9	1.2	0.9	18.4	2.3	58.6		0.5	0.8	0.6	6.1			0.5	0.3			
Lw-55a	*Loktanella* sp.																	100			
MDLw-58	*Dinoroseobacter* sp.																	5.4	3.9	2.5	88.2
Sediment																					
SK013	*Shimia* sp.																		100		
SK032	*Huaishuia* sp.													81.7	18.3						
SK040	*Phaeobacter* sp.		5.8	3.7	1.4	7.3	4.4						4.8	64.9		3.5		4.1			
Water Column																					
SK038	*Sulfitobacter* sp.													100							

^a^ relative amounts of AHLs for each strain in %. No AHLs detected in *Puniceibacterium* sp. Lw-III1a, *Sulfitobacter* sp. B15 G2, *Pseudooceanicola* sp. Lw-13e, *Roseobacter* sp. B14, *Loktanella* sp. SK033, *Phaeobacter* sp. N05I, *Sulfitobacter* sp. B14 27, A12, D4 55, SK012, *Citreicella* sp. Lw-41a, *Roseovarius pelophilus* G5II, *Tateyamaria pelophila* SAM4, *Pseudoruegeria* sp. SK021.

**Table 2:** Activity of synthetic AHLs in quorum sensing experiments with the sensor strains *E.coli* MT102 (pJBA132) and *P. putida* F117 (pRK-C12). Maximum fold induction.

AHL^a^	MT102	F117
2*E*,4*E*-C12:2 (**5**)	1.00 ± 0.01	81.22 ± 1.26
2*E*,5*Z*-C12:2 (**11**)	1.01 ± 0.01	78.03 ± 2.05
3*E*,5*Z*-C12:2 (**13**)	1.03 ± 0	73.64 ± 2.34
2*E*,11*Z*-C18:2	1.02 ± 0.01	1.32 ± 0.04
3-oxo-C8:0	13.41± 0.49	30.46 ± 2.79
C6:0	17.09 ± 1.04	
C8:0	10.24 ± 0.55	
3-oxo-C12:0		77.75 ± 2.51
Z9-C16:1		72.37 ± 2.47

^a^ The final concentration of each tested compound was 10 µM.

**Table 3:** Bacterial isolates with GenBank accession number, genus affiliation and origin.

Strain	GenBank Acc No (16S)	Genus Affiliation	Strain Origin/Host	Location
Lw-III1a	KM268064	*Puniceibacterium* sp.	*Fucus spiralis*	Neuharlingersiel
B15 G2	KM268068	*Sulfitobacter* sp.	*Fucus spiralis*	Neuharlingersiel
Lw-13e	KM268063	*Pseudooceanicola* sp.	*Fucus spiralis*	Neuharlingersiel
D12-1.68	KM268065	*Roseovarius* sp.	*Fucus spiralis*	Neuharlingersiel
B14	KM268066	*Roseobacter* sp.	*Fucus spiralis*	Neuharlingersiel
B14 27	KM268072	*Sulfitobacter* sp.	*Fucus spiralis*	Neuharlingersiel
A12	KM268070	*Sulfitobacter* sp.	*Fucus spiralis*	Neuharlingersiel
D3	KC731427	*Loktanella* sp.	*Fucus spiralis*	Neuharlingersiel
D4 55	KM268071	*Sulfitobacter* sp.	*Fucus spiralis*	Neuharlingersiel
Lw-22	KM268073	*Octadecabacter* sp.	*Fucus spiralis*	Neuharlingersiel
D4 50	KM268074	*Octadecabacter* sp.	*Fucus spiralis*	Neuharlingersiel
Lw-26b	KM268054	*Loktanella* sp.	*Fucus spiralis*	Neuharlingersiel
D15 40	KM268056	*Loktanella* sp.	*Fucus spiralis*	Neuharlingersiel
Lw-55a	KM268057	*Loktanella* sp.	*Fucus spiralis*	Neuharlingersiel
MDLw-58	KM268059	*Dinoroseobacter* sp.	*Fucus spiralis*	Neuharlingersiel
Lw-41a	KM268061	*Citreicella* sp.	*Fucus spiralis*	Neuharlingersiel
N05I	AJ968647	*Phaeobacter* sp.	German North Sea Coast	Tidal-flat sediment
G5II	AJ968650	*Roseovarius pelophilus*	German North Sea Coast	Tidal-flat sediment
SAM4	AJ968651	*Tateyamaria pelophila* ^a^	German North Sea Coast	Tidal-flat sediment
SK012	HG423260	*Sulfitobacter* sp.	Danish North Sea	sediment
SK013	LAJH00000000	*Shimia* sp. ^b^	Norwegian North Sea	sediment
SK021	HG423263	*Pseudoruegeria* sp. ^b^	Norwegian North Sea	sediment
SK032	HG423269	*Huaishuia* sp.	German North Sea	sediment
SK033	HG423270	*Loktanella* sp.	Norwegian North Sea	sediment
SK040	HG423272	*Phaeobacter* sp.	Danish North Sea	sediment
SK038	HG423271	*Sulfitobacter* sp.	German North Sea	seawater

^a^ [32]; ^b^ [33,34].

## Supplementary Materials

The following are available online at http://www.mdpi.com/1660-3397/17/1/20/s1, Figure S1: Mass spectrum of natural compound **B**. Figure S2: ^1^H NMR and ^13^C NMR spectrum of 2*E*,5*Z*-C12:2-HSL (**11**). Figure S3: ^1^H NMR and ^13^C NMR spectrum of 3*E*,5*Z*-C12:2-HSL (**13**). Figure S4: ^1^H NMR and ^13^C NMR spectrum of 2*E*,4*E*-C12:2-HSL (**5**).
