# Supplementary material for: An Unprecedented Medium-Chain Diunsaturated N-acylhomoserine Lactone from Marine Roseobacter Group Bacteria
Source: Mar Drugs. 2018 Dec 31;17(1):20. doi: 10.3390/md17010020 (PMC6356624; doi:10.3390/md17010020)
Supplement: Supplementary file 1 [file marinedrugs-17-00020-s001.pdf]

marine drugs

Supplementary Materials

An Unprecedented Medium-Chain Diunsaturated *N*-acylhomoserine Lactone from Marine *Roseobacter* Group Bacteria

Lisa Ziesche [1], Laura Wolter [2], Hui Wang [3], Thorsten Brinkhoff [2], Marion Pohlner [2], Bert Engelen [2], Irene Wagner-Döbler [3] and Stefan Schulz [1,*]

1 Institute of Organic Chemistry, Technische Universität Braunschweig, Hagenring 30, 38106 Braunschweig, Germany; l.ziesche@tu-braunschweig.de (L.Z.)
2 Institute for Chemistry and Biology of the Marine Environment, University of Oldenburg, Carl-von-Ossietzky-Straße 9-11, 26111 Oldenburg, Germany; laura.wolter@uni-oldenburg.de (L.W.); thorsten.brinkhoff@icbm.de (T.B.); marion.pohlner@uni-oldenburg.de (M.P.); bert.engelen@uni-oldenburg.de (B.E.)
3 Helmholtz Centre for Infection Research, Department of Medical Microbiology, Group Microbial Communication, Inhoffenstr. 7, 38124 Braunschweig, Germany; Hui.Wang@helmholtz-hzi.de (H.W.); Irene.Wagner-Doebler@helmholtz-hzi.de (I.W.-D.)
* Correspondence: stefan.schulz@tu-braunschweig.de; Tel.: +49-531-391-5272

NMR and Mass Spectra

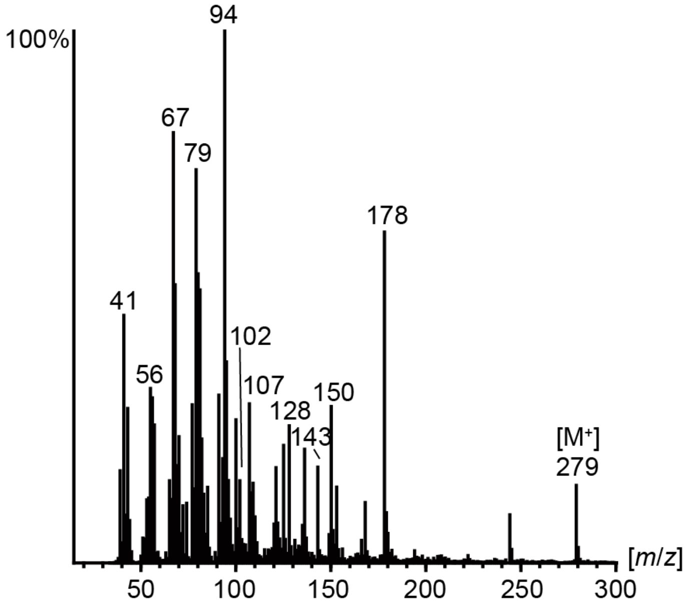

Figure S1. Mass spectrum of natural compound **B**.

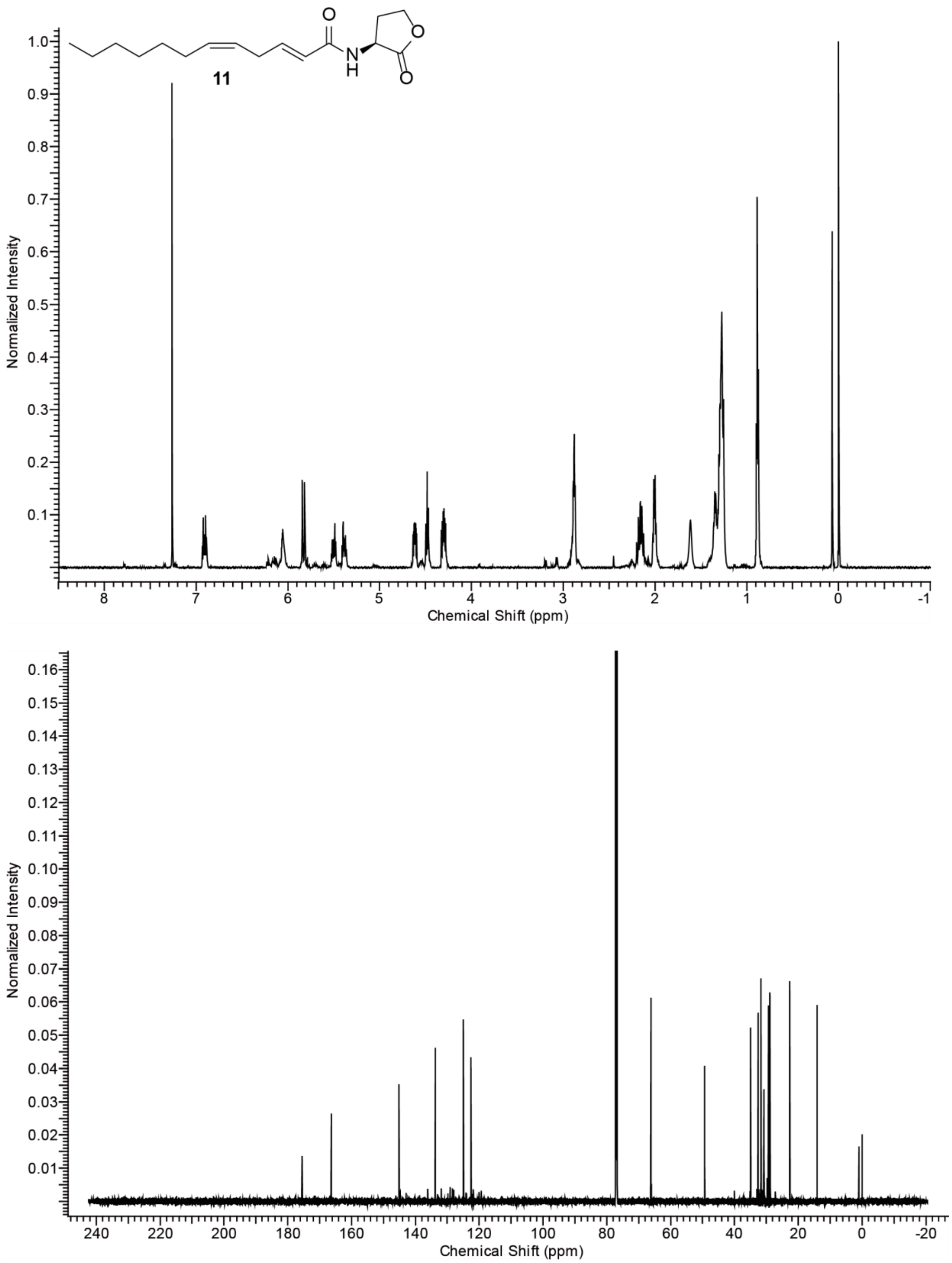

Figure S2. ^{1}H-NMR and ^{13}C-NMR spectrum of *N*-((2*E*,5*Z*)-2,5-dodecadienoyl)homoserine lactone (2*E*,5*Z*-C12:2-HSL, **11**).

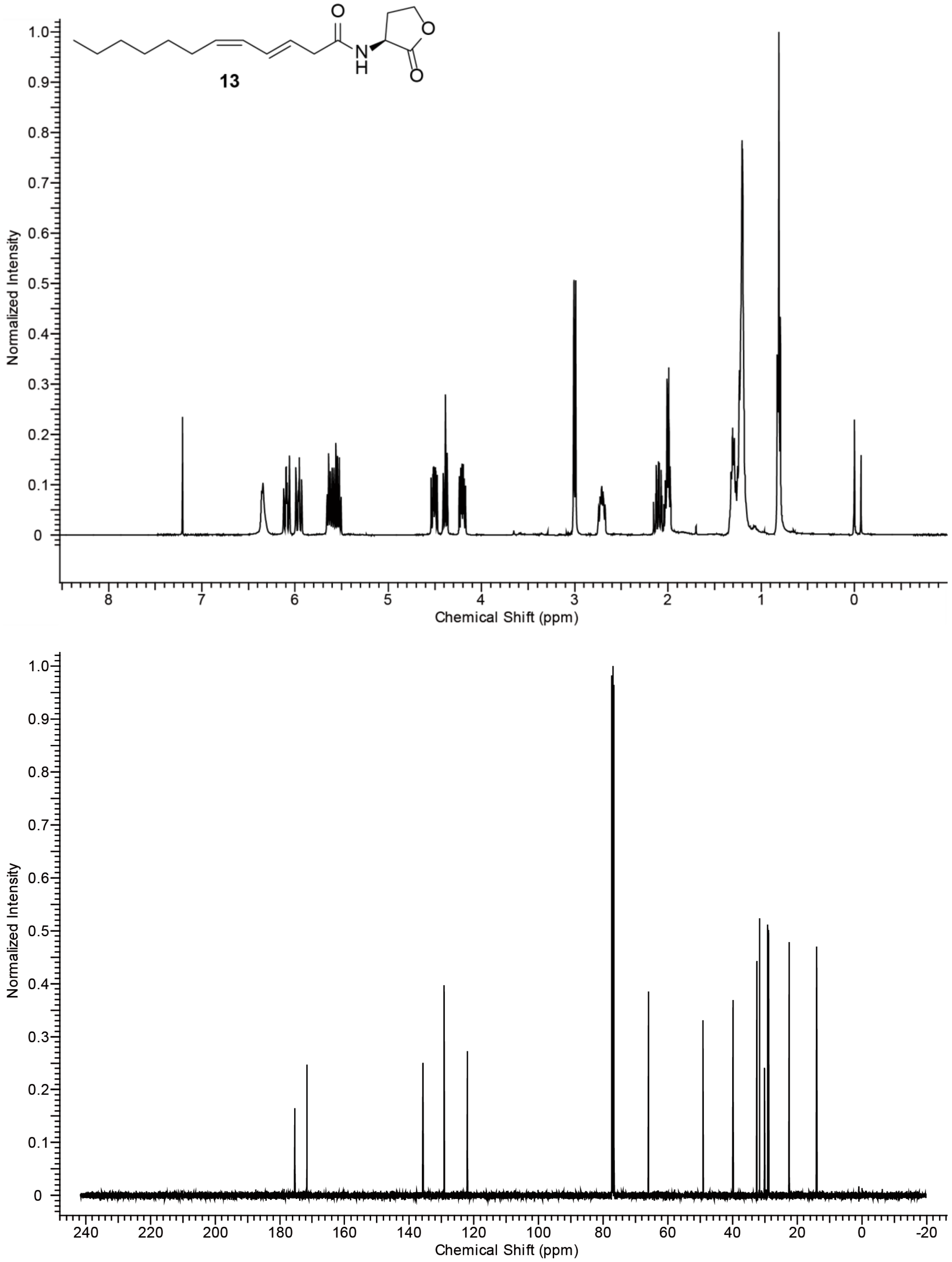

Figure S3. ^{1}H-NMR and ^{13}C-NMR spectrum of *N*-((3*E*,5*Z*)-3,5-dodecadienoyl)homoserine lactone (3*E*,5*Z*-C12:2-HSL, **13**).

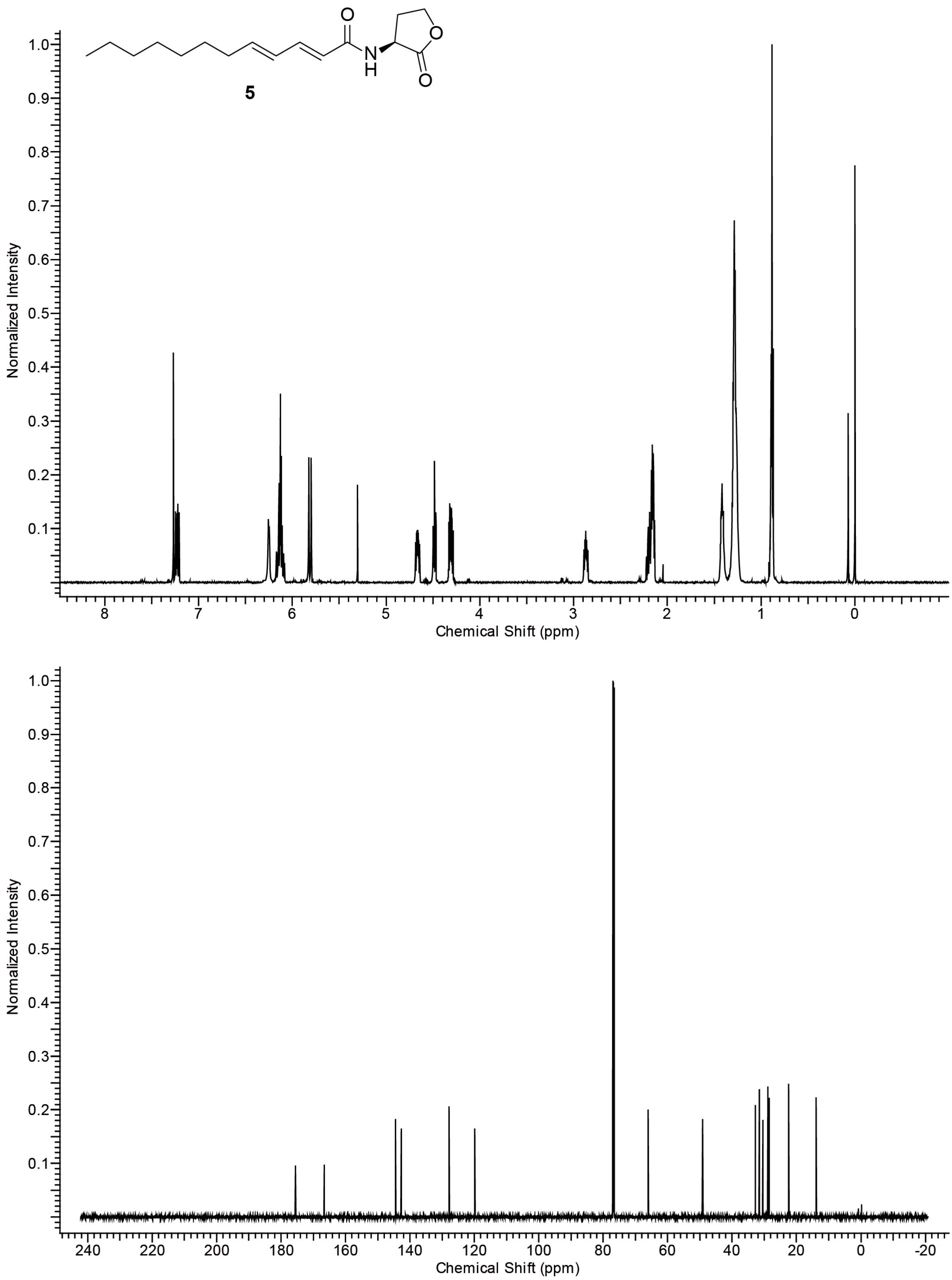

Figure S4. ^{1}H-NMR and ^{13}C-NMR spectrum of *N*-((2*E*,4*E*)-2,4-dodecadienoyl)homoserine lactone (2*E*,4*E*-C12:2-HSL, **5**).
